# *Trans*-Regulation of Mouse Meiotic Recombination Hotspots by *Rcr1*

**DOI:** 10.1371/journal.pbio.1000036

**Published:** 2009-02-17

**Authors:** Emil D Parvanov, Siemon H. S Ng, Petko M Petkov, Kenneth Paigen

**Affiliations:** Center for Genome Dynamics, The Jackson Laboratory, Bar Harbor, Maine, United States of America; National Cancer Institute, United States of America

## Abstract

Meiotic recombination is required for the orderly segregation of chromosomes during meiosis and for providing genetic diversity among offspring. Among mammals, as well as yeast and higher plants, recombination preferentially occurs at highly delimited chromosomal sites 1–2 kb long known as hotspots. Although considerable progress has been made in understanding the roles various proteins play in carrying out the molecular events of the recombination process, relatively little is understood about the factors controlling the location and relative activity of mammalian recombination hotspots. To search for *trans*-acting factors controlling the positioning of recombination events, we compared the locations of crossovers arising in an 8-Mb segment of a 100-Mb region of mouse Chromosome 1 (Chr 1) when the longer region was heterozygous C57BL/6J (B6) × CAST/EiJ (CAST) and the remainder of the genome was either similarly heterozygous or entirely homozygous B6. The lack of CAST alleles in the remainder of the genome resulted in profound changes in hotspot activity in both females and males. Recombination activity was lost at several hotspots; new, previously undetected hotspots appeared; and still other hotspots remained unaffected, indicating the presence of distant *trans*-acting gene(s) whose CAST allele(s) activate or suppress the activity of specific hotspots. Testing the activity of three activated hotspots in sperm samples from individual male progeny of two genetic crosses, we identified a single *trans*-acting regulator of hotspot activity, designated *Rcr1*, that is located in a 5.30-Mb interval (11.74–17.04 Mb) on Chr 17. Using an Escherichia coli cloning assay to characterize the molecular products of recombination at two of these hotspots, we found that *Rcr1* controls the appearance of both crossover and noncrossover gene conversion events, indicating that it likely controls the sites of the double-strand DNA breaks that initiate the recombination process.

## Author Summary

Recombination is an essential aspect of meiosis, ensuring proper contact and exchange of genetic material between homologous parental chromosomes, as well as their subsequent segregation to produce haploid gametes. In humans and mice, recombination events are located at preferential sites termed hotspots, whose placement and activity are tightly regulated. We have now identified a hotspot-regulating locus in mammals, *Rcr1*, that simultaneously controls the locations of multiple hotspots. The discovery of *Rcr1* indicates the existence of a newly emerging class of genes important in the recombination processes. Gaining further insights into their function may contribute to a better understanding of genetic factors underlying human fertility and evolution.

## Abstract 3

*Rcr1* is identified as a*trans*-regulator of meiotic recombination hotspots that appears to act at the initiation of recombination and that maps to a 5.3-megabase region on mouse Chromosome 17.

## Introduction

Meiotic homologous recombination is responsible for generating genetic variety among offspring as well as ensuring accurate chromosome segregation during meiotic cell divisions. The process of recombination is initiated by the formation of DNA double-strand breaks (DSBs) created by the highly conserved topoisomerase IV–like protein SPO11 [1]. These DSBs provide the sites at which chiasmata and crossovers form, events that are necessary for proper chromosome alignment and segregation in Meiosis I. When the repair of a DSB involves a homologous chromatid, the outcome can be recognized genetically as either a reciprocal exchange of genetic information between the homologous chromatids (a crossover [CO]), or alternatively as the unidirectional acquisition of genetic information by the initiating chromatid from its non-initiating partner (a noncrossover [NCO], sometimes referred to as a gene conversion) [2,3]. Studies in Saccharomyces cerevisiae show that COs and NCOs are the preferred outcomes of two alternative pathways in meiotic recombination, COs being predominantly produced by the Double-Strand Break Repair (DSBR) pathway, and NCOs predominantly produced by the Synthesis-Dependent Strand-Annealing (SDSA) pathway [4,5].

Importantly, in all organisms, meiotic recombination does not occur at uniform rates along chromosomes. In both yeast and mammals—the most extensively studied cases—recombination rates vary considerably along the length of a chromosome [6–10]. When examined at high resolution, the great majority of recombination, possibly all, occurs in restricted regions, termed hotspots, that are typically 1–2 kb long in humans and mice [11,12].

In contrast to the considerable body of information describing the participation of a variety of proteins in the overall processes of recombination, relatively little is presently understood about the factors determining the chromosomal locations and relative activity of recombination hotspots. In yeast, hotspots have been classified into three groups based on their presumed activation requirements. Activation of “α” hotspots requires transcription factors; the “β” hotspots require the presence of nuclease-sensitive chromatin (a necessary, but not sufficient, condition); and the “γ” hotspots are dependent on the G+C content of DNA (reviewed in [13]). Many yeast hotspots can be assigned to more than one class due to multiple mechanisms involved in the initiation of recombination. There is no obvious consensus sequence defining hotspots in either S. cerevisiae or Schizosaccharomyces pombe with the exception of the class of hotspots in S. pombe, which have an 18-bp consensus sequence containing the CRE-heptameric cyclic AMP response element ATGACGT [14–16]. However, this sequence accounts for only a minority of hotspots in *S. pombe*; the defining elements of the remainder are unknown. A 13-bp consensus sequence has been identified that is present in 41% of human hotspots [17]; although this sequence is highly enriched in hotspots, its presence alone is not sufficient to initiate hotspot activity, suggesting that other presently unknown factors are also required. This 13-bp sequence also has the interesting property of serving as a site of spontaneous DNA breakage in mitochondrial DNA.

That the location of a hotspot is not determined simply by its internal DNA sequence was shown by DSB mapping experiments in S. cerevisiae. The DSBs initiating recombination preferentially occurred within a window of 100–500 bp near the center of the hotspot [18–21]. However, replacement of these preferred sites for DSB formation did not eliminate DSB formation there, and DSBs now occurred at the replacement sequence [22,23].

*Trans*-acting factors controlling hotspot activation have been identified in several cases in yeast. Binding of the *ATF1/PCR1* transcription factor is required for activity of the aforementioned *ADE6-M26* hotspot in S. pombe [24], and activity of the *HIS4* hotspot in S. cerevisiae requires binding by the transcription factors *BAS1*, *BAS2*, and *RAP1* [25] and *GCN4* [26]. A more extensive analysis of the effect of *BAS1* has shown that loss of this protein can either reduce or increase the recombination activity of a number of S. cerevisiae hotspots [27].

Recent data indicate that such *trans*-acting factors may act through posttranslational modifications of histones with attendant nucleosome rearrangements. For example, in the *MAT2-MAT3* cold region in S. pombe, the cooperative action of histone deacetylases and histone methyltransferases contribute to recruitment of heterochromatin proteins, keeping the region both transcriptionally and recombinationally silent [28] and directing recombination to the adjacent mating-type locus [29]. Regulation of recombination by histone methyltransferases has also been shown in S. cerevisiae [30,31] and Caenorhabditis elegans [32], and histone H2B ubiquitination has been shown to play a role in DSB formation, by recruitment and/or stabilization of DSB-initiating factors through *RAD6-BRE1* [33]. The most detailed analysis of the influence of chromatin modifications on meiotic recombination has been achieved for the *ADE6-M26* hotspot in S. pombe, at which a set of histone acetyltransferases and ATP-dependent chromatin remodeling factors alter chromatin structure, regulating both transcription and recombination [34].

In contrast to what is known in yeast, we know considerably less about possible *trans*-acting factors influencing the location and relative activity of mammalian hotspots, although there is now evidence that such factors exist. The *MSTM 1a* and *1b* hotspots in humans vary considerably in activity among individual males even when they share the same haplotype within and around the hotspots themselves [35], suggesting control by either *trans*-acting factors or very distant *cis*-acting factors; and in mice, Baudat and de Massy [36] have shown that initiation of recombination at the *Psmb9* hotspot on chromosome 17 (Chr 17) requires the presence of a *trans*-acting gene located some distance proximal to the site of the hotspot.

To systematically explore the possible existence and identity of *trans*-acting factors controlling hotspot specificity in mammals, we have taken advantage of the possibilities inbred mouse strains provide for genetic analysis and compared the recombination maps generated along a region of mouse Chr 1 when this region was always heterozygous C57/BL6 (B6) × CAST/EiJ (CAST), and the rest of the genome either did or did not carry CAST alleles. In doing so, we found hotspots whose activity was either dependent upon, or suppressed by, the presence of CAST alleles at distant loci, whereas other hotspots were unaffected. Assaying the activity of specific hotspots in the sperm of males segregating in genetic crosses, we found that the activity of several hotspots depended on a single Mendelian factor we have designated *Recombination regulator 1* (*Rcr1*) that maps to a 5.30-Mb region on proximal Chr 17. Molecular assays of individual products of recombination at these hotspots indicated that *Rcr1* acts to control the initiation of recombination rather than the choice between the CO and NCO pathways of the recombination process. In their accompanying paper, Grey et al. [37] describe a similar *trans*-acting locus controlling the appearance of both CO and NCO gene conversions at the *Psmb9* hotspot on Chr 17 of the mouse as well as elsewhere in the genome.

## Results

### Transactivation and Suppression of Specific Hotspots

**Figure 1:**
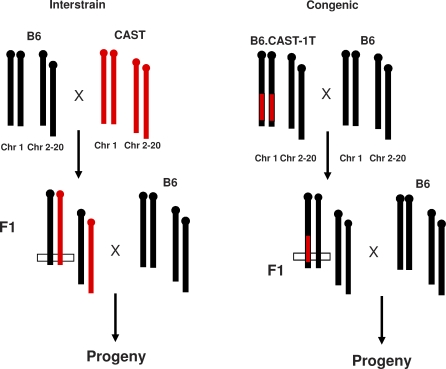
Crosses Used for Recombination Map on Chr1 Crosses of B6xCAST (interstrain) and B6xB6.CAST-1T (congenic): B6 sequences are black; CAST sequences are red. The region within which recombination was measured is boxed; the crossovers are detected in the final progeny.

The existence of *trans*-acting genes became apparent when comparing the recombination maps obtained by two genetic crosses involving the B6 and CAST mouse strains, which were chosen for their genetic diversity. In the first cross, hereafter referred to as the interstrain cross, B6 mice were mated to CAST, and the F1 hybrids were backcrossed to B6. In the second cross, hereafter referred to as the congenic cross, B6 were mated to B6.CAST-1T, a congenic strain carrying 100 Mb of CAST DNA sequences from distal Chr 1 introgressed into C57BL/6J (see Materials and Methods for details of this strain); the resulting F1 hybrids were then backcrossed to B6. In both crosses, the F1 hybrids shared the same heterozygous 100-Mb segment on Chr 1. The fundamental difference was the presence of CAST alleles in the remainder of the genome in the interstrain B6xCAST F1 animals and their absence in congenic B6xB6.CAST-1T F1 mice, which are homozygous B6 outside the 100-Mb Chr 1 region (Figure 1).

In both crosses, recombination was tested in an 8-Mb region (183.5–191.5 Mb, National Center for Biotechnology Information [NCBI] build 36) located within the 100-Mb heterozygous B6/CAST region. The B6xCAST recombination map utilized the products of 6,028 meioses occurring in F1 animals (3,002 offspring of female F1 and 3,026 offspring of male F1) and was part of the entire map of Chr 1 described previously [10]. Among these offspring, 735 contained a single CO event in the 8-Mb region (264 arising in female F1s and 471 in male F1s), producing a total sex-averaged map length of 12.2 cM (8.8 cM in females and 15.6 cM in males), or 1.52 cM/Mb (1.1 cM/Mb and 1.95 cM/Mb in females and males, respectively). The B6xB6.CAST-1T map utilized 2,083 meioses (1,173 offspring of female F1 and 910 offspring of male F1), of which 175 meioses provided a single CO event in the 8-Mb region (83 from females and 92 from males). The sex-averaged map length of the region in this cross was 8.4 cM (7.1 cM in females and 10.1 cM in males), or 1.05 cM/Mb (0.89 cM/Mb and 1.26 cM/Mb in females and males, respectively). To test for any possible effect of genomic imprinting on recombination rates, half of the offspring of each cross were derived from F1 animals in which the dam was B6 and the sire was CAST or B6.CAST-1T, and the other half were derived from a reciprocal parental combination; there were no significant differences in hotspot locations between these reciprocal crosses.

**Figure 2:**
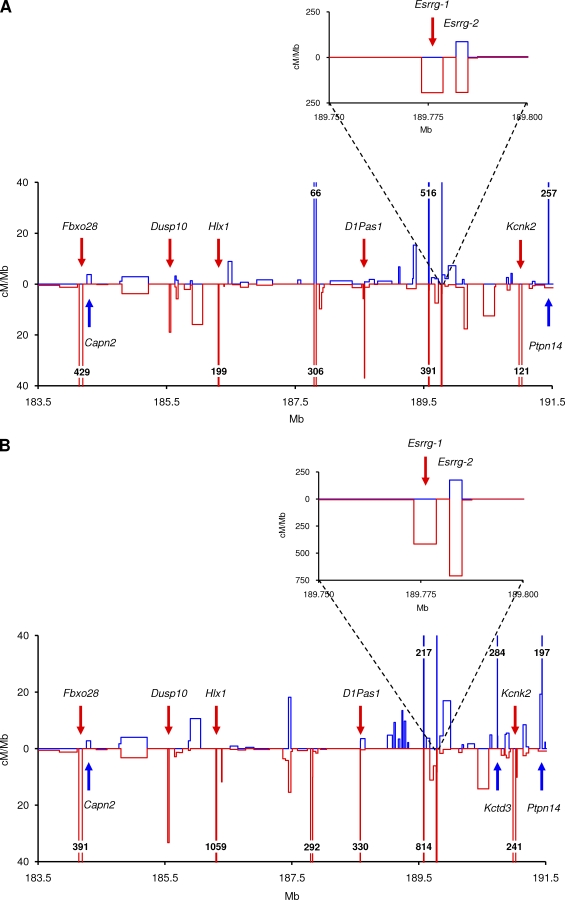
Recombination Map of the Chr 1 Region between 183.5 and 191.5 Mb (A) Comparison between the female maps of B6xB6.CAST-1T (blue line) and B6xCAST (red line). (B) Comparison between the male maps of the two crosses using the same colors. The maps of B6xCAST are taken from previous experiments [10]. Recombination rates are expressed in centimorgans per megabase for the intervals between adjacent markers. Hotspots included in Table 1 are shown with their names and positions indicated by arrows. Red arrows mark hotspots where recombination was present only in the B6xCAST cross; blue arrows mark regions where recombination was present only in the B6xB6.CAST-1T cross. The numbers show actual centimorgans per megabase values for hotspots exceeding the limits of the figure. The insets expand the map in the 50-kb region between 189.750 and 189.800 Mb; the red arrow marks the *Esrrg-1* hotspot that is active only in the B6xCAST cross.

The COs occurring in the 8-Mb region of Chr 1 were mapped to hotspot-level resolution using the same markers for both crosses. Figure 2 presents the female and male recombination maps obtained. Although most regions showed similar activity in both crosses, there were nine regions, indicated by arrows, where the results differed dramatically; six hotspots disappeared in the congenic cross (*Fbxo28*, *Dusp10*, *Hlx1*, *D1Pas1*, *Esrrg-1*, and *Kcnk2*, named after their closest genes), and three regions that were devoid of recombination in the interstrain cross contained new, active hotspots in the congenic cross (*Capn2*, *Kctd3*, and *Ptpn14*). Three hotspots in the interstrain cross (*Hlx1*, *Esrrg-1*, and *Kcnk2*) and one in the congenic cross (*Kctd3*) showed statistically significant sex differences. In no case did we find a hotspot whose activity depended on a CAST allele in one sex, but not the other, in a statistically significant manner. Particularly notable were the twin hotspots *Esrrg-1* and *Esrrg-2*, which in the interstrain cross are separated by less than 5 kb and are significantly more active in males than in females. Only the hotspot proximal to the centromere, *Esrrg-1*, disappeared in the congenic cross; the other, *Esrrg-2*, remained active, although with reduced activity (inserts on Figure 2A and 2B). The probabilities of these results being observed by chance are very low (Table 1). One of the regions (188.7–189.1 Mb) in which recombination appeared in the congenic cross stretched across several markers, indicating that this region contains several distinct, activated hotspots.

**Table 1:**
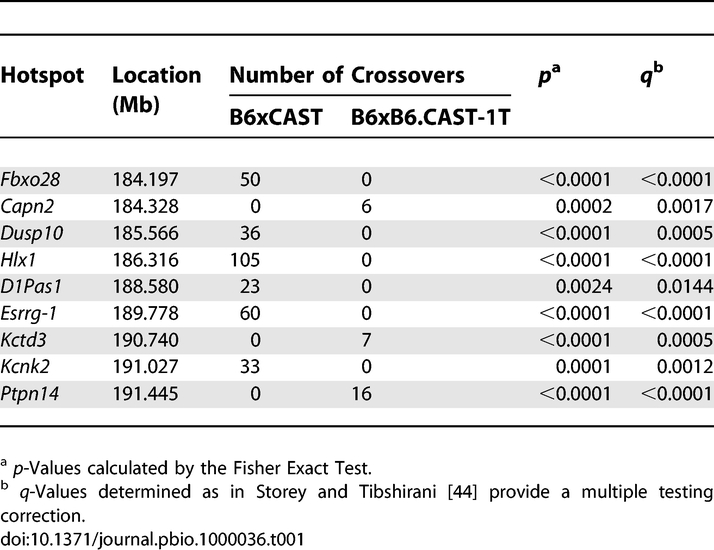
Recombination Hotspots Active in Only the Interstrain or the Congenic Backcross

The rates at regions with centromere-proximal ends at 185.220, 185.900, and 187.485 Mb were similar, and others like those at 187.827, 189.584, 189.785, and 190.001 Mb were active in both crosses but with different activities.

It is apparent from these results that the products of CAST alleles of distant loci can activate or suppress the activity of individual hotspots without affecting other hotspots in the same chromosomal region.

### Mapping *Rcr1*, the *Trans*-Acting Locus

**Figure 3:**
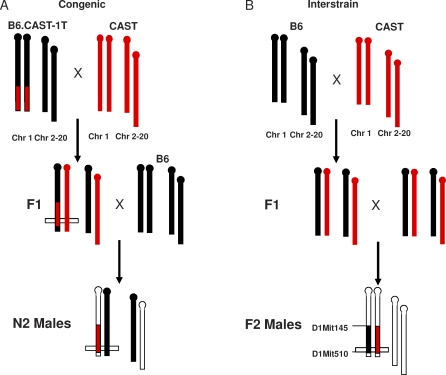
Crosses for Mapping of *Trans*-Acting Genes (B6.CAST-1TxCAST)xB6 (A) and B6xCAST (B) crosses used for mapping *trans*-acting genes. B6 sequences are in black, and CAST in red. The region where the recombination is studied is boxed. Segregating loci are located in the white regions. Note that any recombination within the congenic region between the B6.CAST-1T and CAST chromosomes does not alter the allelic composition of this region.

**Figure 4:**
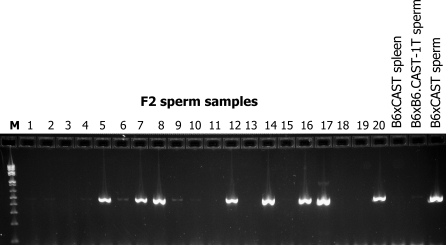
Allele-Specific PCR for Hotspot *Hlx1* Gel electrophoresis of F2 samples from the interstrain cross after two rounds of allele-specific PCR. M is a 100-bp ladder; 1–20 are F2 samples; the last three lanes contain negative and positive controls as described.

Two mapping crosses were used in searching for *trans*-acting genes regulating recombination at specific hotspots. In the first cross, F1 females derived from a cross between the B6.CAST-1T congenic strain and CAST were backcrossed to B6 (Figure 3A). The progeny of this cross were all heterozygous B6/CAST at the 100-Mb congenic region and segregated CAST alleles in the remainder of the genome. The advantages of this cross were that it allowed the detection of any relevant X-linked genes; that all male progeny were informative, and that any CAST alleles were always present in the heterozygous condition, as they were in the F1 animals where the differences in hotspot activity were originally detected. A total of 211 male animals from this cross were individually phenotyped for activity of three hotspots *Hlx1* (186.316 Mb, located 110 kb away from the gene's 3′-end), *Esrrg-1* (189.778 Mb, together with its neighbor *Esrrg-2* located in intron 3–4 of *Esrrg*) and *Kcnk2* (191.027 Mb, located in intron 2–3 of its namesake gene). Phenotyping was carried out using allele-specific sperm DNA assays (see Materials and Methods); these assays gave a plus/minus phenotype for each of the three hotspots; a hotspot was either active or missing from genetically segregating mice (Figure 4). Unfortunately, because suitable single nucleotide polymorphism (SNP) combinations were not available, it was not possible to develop nested PCR assays for any of the three hotspots suppressed by the presence of *trans*-acting CAST alleles.

To address possible dosage effects of CAST alleles on recombination activity, a second cross was carried out in which B6xCAST F1 animals were mated together; the half of the resulting F2 male progeny that were heterozygous at the distal part of Chr 1 (including the entire congenic region between the microsatellite markers *D1Mit145* located at 169.132 Mb and *D1Mit510* located at 194.118 Mb (Figure 3B) were tested for hotspot activity. As with the first mapping cross, these animals were heterozygous for the distal part of Chr 1, but segregated both B6 and CAST alleles in either the homozygous or heterozygous state in the remainder of the genome. In all, 98 animals from this F2 cross representing 196 meioses were phenotyped.

Phenotyped animals from both crosses were genotyped with 165 SNP markers spaced across the genome, ensuring 20-Mb resolution (Table S1). The genotyping and phenotyping data were analyzed by the R/QTL-based software package J/qtl (http://research.jax.org/faculty/churchill/software/Jqtl/index.html).

**Figure 5:**
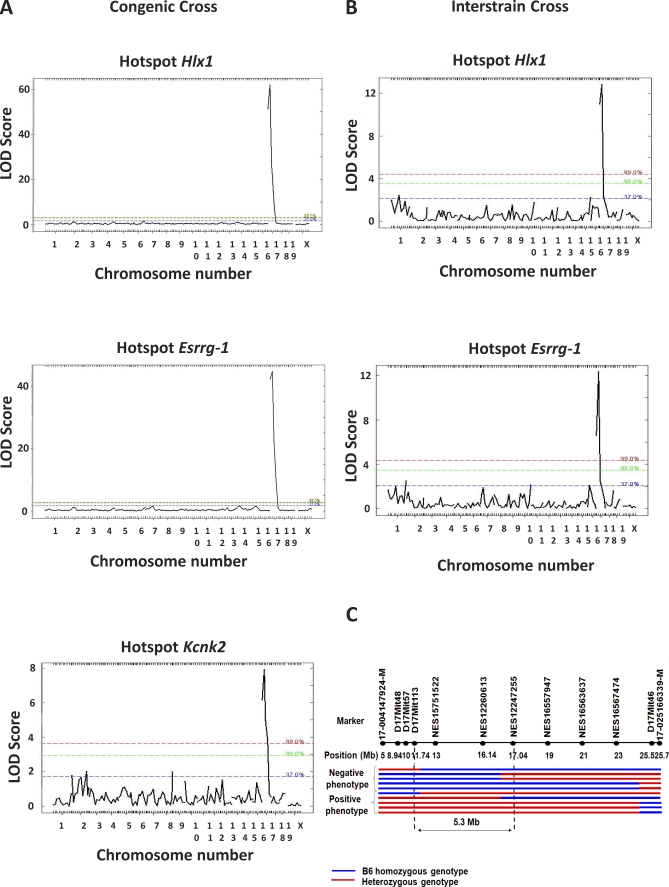
QTL Analysis of Phenotyping and Genotyping Data (A) A total of 211 samples from the first mapping and (B) 98 samples from the second mapping cross were used in the analysis. The LOD scores are the result of 10,000 permutations per analysis. (C) Further mapping on Chr 17 of recombinants in the interval 5–25 Mb, including eight recombinants from the congenic backcross and two from the interstrain cross. The borders of the critical region within which *Rcr1* must lie are marked with dashed vertical lines.

The results showed strong linkage between hotspot activities and a single interval on proximal Chr 17 located between 5 and 25 Mb, with LOD scores above 30 for hotspots *Hlx1* and *Esrrg-1* and around 8 for hotspot *Kcnk2* in the congenic backcross (Figure 5A), and above 10 for hotspots *Hlx1* and *Esrrg-1* in the interstrain cross (Figure 5B). The two crosses produced identical map locations, providing evidence that CAST alleles in either homozygous or heterozygous condition activate recombination at the analyzed hotspots. No other chromosome location showed significant linkage in either cross. The reason for the lower LOD scores with the *Kcnk2* hotspot is the lower efficiency of the nested PCR assay for this hotspot, which although it never gave a false-positive result with control DNA samples, did not always give a positive result with samples known to contain COs.

We have designated the Chr17 locus *Recombination regulator 1* (*Rcr1*). To further refine the location of *Rcr1*, all of the crossovers occurring between 5 and 25 Mb on Chr 17 were typed for a combination of microsatellite and SNP markers (Figure 5C). The left border of the critical interval was located between 11.74 Mb (*D17Mit113*) and 13.01 Mb (NES15751522), and the right border was located between 16.14 Mb (NES12260613) and 17.04 Mb (NES12247255), showing that *Rcr1* must lie in the 5.30-Mb interval between 11.74 and 17.04 Mb on Chr17.

### *Rcr1* Controls the Initiation Steps of the Recombination Process

We tested whether *Rcr1* controls the earlier stages of recombination process, between the initiation of DSB and the formation of recombination intermediates, or the later decision to process the recombination intermediates into either COs or NCOs. If *Rcr1* acts early in recombination, it should control the appearance of both COs and NCO gene conversions at susceptible hotspots. If, however, it acts on the choice between CO and NCO pathways as alternative outcomes of repairing the DSBs that initiate recombination, we would expect to see persistence of NCOs at susceptible hotspots in the absence of the *Rcr1* CAST allele.

A cloning assay counting the number of COs and NCOs at individual hotspots in F1 sperm DNA [38] was used to make this distinction. In essence, the region containing the hotspot was amplified from sperm DNA using primers common to both B6 and CAST. The amplified product was then cloned into E. coli such that the mammalian DNA in each clone is derived from a single strand of an individual sperm, and the individual clones were genotyped (see Material and Methods). This assay was feasible for the *Hlx1* and *Esrrg-1* hotspots, in which the availability of suitable internal markers facilitated the detection of both COs and NCOs. In B6/CAST F1 sperm, in which these hotspots are expected to be active, both COs and NCOs were present at the two hotspots, but in B6/B6.CAST-1T F1 sperm, in which the hotspots are expected to be inactive, NCOs as well as COs were entirely absent from these hotspots, suggesting that *Rcr1* controls the initiation of recombination (Table 2).

**Table 2:**
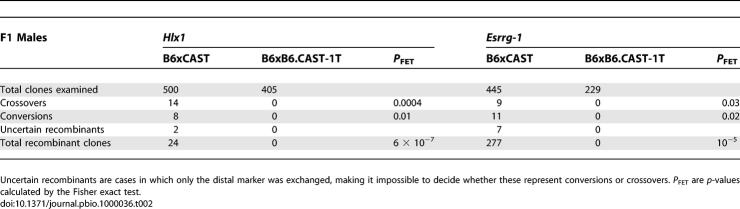
Results of the *E. coli* Cloning Assay for Crossing Over and Conversion at Hotspots *Hlx1* and *Esrrg-1*

## Discussion

Our major findings are, first, that the recombination activity of several hotspots on mouse Chr 1 is either activated or suppressed by the presence of CAST allele(s) of distant *trans*-acting loci; second, that activation of several of the hotspots is controlled by a locus, *Rcr1*, located within a 5.30-Mb window on proximal Chr 17; and third, that *Rcr1* exerts its effect at the initiation of recombination, prior to the CO–NCO choice.

*Rcr1* does not have a generalized effect on recombination rates as many of the hotspots within the 8-Mb segment of Chr 1 mapped here were not affected by the presence versus absence of a CAST allele at this locus; moreover, the number of regions exhibiting greater than 0.1% recombination in the congenic cross (21) is similar to the number of such regions (20) in the interstrain cross across (Figure 2). Although the total recombination rate across this 8-Mb region is somewhat lower in the congenic cross compared to the interstrain cross, presumably due to the loss in the congenic cross of several hotspots that were highly active in the interstrain cross, this appears to be a localized effect as the overall recombination rate across the larger interval 169–193 Mb was the same in both crosses (unpublished data).

Some of the hotspots in the 8-Mb tested region remained active in both crosses, leaving open the question of what controls their activity. Presumably, these hotspots are activated either by the B6 allele of *Rcr1*, which was always present, or by other *trans*-acting genes. Previous observations suggest that there are approximately 13,500–14,000 hotspots across the mouse genome active in the same B6xCAST cross used in this study [10], and the known variation in hotspot usage among different mouse crosses suggests a potentially even higher number for the species as a whole. This is true for the human genome as well, which is estimated to contain an even larger number of hotspots [9]. Given these high numbers, it is unlikely that each hotspot is controlled by its specific *trans*-acting gene, and it is more likely that, as in the present case, a *trans*-acting gene controls a family of hotspots. However, the only prior indications that such families might exist were the finding that the 13-bp consensus sequence CCNCCNTNNCCNC occurs in 41% of human hotspots ([17] and the existence of the *ATF1.PCR1* transcription factor of S. pombe which binds to an 18-bp consensus sequence, effecting a chromatin reorganization in the region and activating recombination [16].

It is interesting that although activation of the *Hlx1* hotspot requires the presence of a CAST allele at *Rcr1*, former investigations showed that recombination at this hotspot initiates almost three times more frequently on the B6 chromatid then on the CAST one [10]. The origin of this seemingly contradictory effect may lie in the so-called “hotspot paradox” [39], which postulates that whenever there are *cis*-acting sequences influencing the activity of hotspots, because in the process of recombination, the more active initiating chromatid acquires the DNA sequence of its less active partner more frequently than the reverse, hotspots will slowly degrade their activity over time as any mutations diminishing activity accumulate in the population. In the case of *Hlx1*, which is activated by the CAST allele present in *Mus musculus castaneus*, but not by the B6 allele present in *M. m. domesticus*, it may be that over evolutionary time, the *Hlx1* haplotype has been under selection pressure for diminished activity in *M. m. castaneus* but not *M. m. domesticus*, leaving the B6 *Hlx1* haplotype closer to the more active primordial sequence. Although indirect, this may be the first experimental evidence that the hotspot paradox does operate over evolutionary time.

These results with hotspot *Hlx1* demonstrate that activity of a hotspot can be determined by the interaction of a *trans*-acting factor with *cis*-acting DNA sequences on each chromatid. A similar interaction between *cis*- and *trans*-acting elements has been reported at the *Psmb9* hotspot on mouse Chr 17 [36]. In their accompanying paper, Grey et al [37] now report that this *trans*-acting element (*Dsbc1*) is located on Chr 17 within a 6.7-Mb region between 10.1 and 16.8 Mb, and affects the distribution of recombination in other regions of Chr 17, Chr 15, and Chr 18, as well as the *Hlx1* hotspot described here. The *Dsbc1* interval overlaps the location of *Rcr1*, suggesting that *Rcr1* and *Dsbc1* are likely to be the same gene or at least members of the same gene family.

The evidence that *Rcr1* acts prior to the choice between processing DSBs into COs or NCOs derives from the fact that both CO and NCO products of recombination at hotspots *Hlx1* and *Esrrg-1* depend upon the presence of a CAST allele at *Rcr1*. If mammalian recombination processes parallel those in the yeast, this choice is made very early in the recombination process [4,5,40]. Given that result, and the difficulty of sustaining any unrepaired DSBs during meiosis, it seems highly likely that *Rcr1* influences the choice of sites for the SPO11 catalyzed DSBs that initiate the recombination process.

At this point, the molecular identity of *Rcr1* is unknown. The closest known phenotypic parallel is the *ADE6-M26* hotspot in S. pombe that is activated via chromatin remodeling mediated by the *ATF1.PCR1* transcription factor. Somewhat less similar is the effect of histone deacetylase *SIR2* ^−/−^ mutants on recombination at multiple sites in S. cerevisiae [41]. In this latter case, the magnitude of the effects and their frequency differ appreciably from what is seen in mice; only 12% of recombination sites were affected, and less than 1% of those affected (<0.1% of total sites) showed greater than 5-fold difference between mutant and wild type. Moreover, the 12% of recombination sites affected at any level tended to be regionally concentrated depending on whether they showed increased or decreased recombination. Mieczkowski et al. [41] drew the reasonable conclusion that *SIR2* likely affects regional chromatin configurations. If *Rcr1* acts by modifying chromatin, it must do so over a very small range as the *Esrrg-1* and *Esrrg-2* hotspots differed markedly in their responses to the presence of an *Rcr1* CAST allele despite being less than 5 kb apart.

Whatever the molecular nature of the *Rcr1* gene product, the existence of such *trans*-acting factors offers a potential means of explaining several presently enigmatic features of mammalian recombination, including varying hotspot activities (the relative affinity of adaptors for their cognate sequences), sex differences in activities of individual hotspots (differential rates of transcription), and the failure to find a consensus DNA sequence that accounts for the specificity of SPO11 cleavage (if SPO11 acts by recognizing the existence of complex between a hotspot and its cognate adaptor, each family of hotspots may have its own, unique consensus sequence).

## Materials and Methods

### Strains and DNA preparation.

C57BL/6J and CAST/EiJ were obtained from the Jackson Laboratory. B6.CAST-1T (rs3022828–rs13476307) was kindly provided by Dr. Wesley Beamer [42].

The Jackson Laboratory is American Association for Laboratory Animal Science (AALAS) accredited, and the Jackson Laboratory Animal Care and Use Committee approved all animal procedures.

### Partially purified DNA was used for genotyping.

The sample preparation was done as described earlier [10]. Sperm phenotyping used DNA from 12-wk-old animals. Sperm DNA was isolated by a modified protocol using the DNeasy tissue kit (Qiagen) as described before [38].

### E. coli cloning assay.

The *E. coli* cloning assay was applied to hotspots *Hlx1* and *Esrrg-1* as described previously [38]. The hotspot sequence was amplified with primers common to both parents, and the DNA fragments were cloned in E. coli such that each colony represents a single DNA strand from the initial meiotic event. Fluorescent SNP genotyping was carried out directly on an aliquot of E. coli cultures grown from each colony in 96-well plates.

### Phenotyping of recombination activity.

Recombination activity was detected by selectively amplifying recombinant DNA fragments at the hotspot sequence of interest. Sperm DNA samples were subjected to two rounds of nested PCR using allele-specific primers in each of the two rounds. The two pairs of primers were oriented in the B–C combination: the proximal pair was specific to B6 alleles flanking the hotspot, and the distal pair to CAST alleles. The primers were PTO-modified at the last three nucleotides of their 3′-end (MWG-Biotech). All primer sequences and positions are summarized in Table S2. The PCR conditions for each tested hotspot were empirically established to ensure that the amplified product is only from the recombinant class (B–C) and not the parental types (B–B and C-–C) (Figure 4). The PCR reaction was performed on an Eppendorf PCR system (Eppendorf AG), using 50-ng initial amount of DNA template and 0.23 mM each dNTP, 0.23 μM of each primer, 1× TITANIUM Taq PCR Buffer, 0.4 U TITANIUM Taq DNA Polymerase (Clontech) for the first round of PCR. The amplified product was diluted 500 times and used for the second allele-specific PCR. The PCR cycling conditions for hotspot *Hlx1* were first round—initial denaturizing step at 94 °C for 4 min, and 45 rounds of 94 °C for 1 min, 63 °C for 40 s, 72 °C for 3 min 20 s, and a final extension at 72 °C for 10 min; second round—initial denaturizing step at 94 °C for 1 min, and 35 rounds of 94 °C for 50 s, 64 °C for 35 s, 72 °C for 1 min, and a final extension at 72 °C for 7 min. The cycling conditions for the *Esrrg-1* hotspot were first round—initial denaturizing step at 94 °C for 2 min, and 30 rounds of 94 °C for 1 min, 63.2 °C for 1 min, 68 °C for 2 min 30 s, and a final extension on 72 °C for 7 min; second round was the same as for hotspot *Hlx1*. For hotspot *Kcnk2*, the cycling conditions were first round—initial denaturizing step at 94 °C for 4 min, and 45 rounds of 94 °C for 1 min, 63 °C for 40 s, 72 °C for 4 min 30 s, and a final extension at 72 °C for 10 min; second round—initial denaturizing step at 94 °C for 4 min, and 35 rounds of 94 °C for 1 min, 64 °C for 30 s, 72 °C for 4 min, and a final extension at 72 °C for 10 min. The amplified product was run through standard 2% agarose gel (Invitrogen) with ethidium bromide and visualized under UV light.

### Genotyping.

Fine mapping of recombination activities in the region of 183.5–191.5 Mb on Chr 1 was carried out using SNP markers and Amplifluor SNPs and the HT FAM-JOE System (Millipore). All markers used in this study and their positions according to NCBI build 36 are summarized in Table S3. For genome-wide association mapping, all progeny were genotyped at 20-Mb resolution using the KASPar genotyping system (KBiosciences). The markers were selected from the Jackson Laboratory genotyping panel [43]. The data were analyzed by SNPviewer2 (KBiosciences). Fine mapping of CAST alleles on Chr 17 was done by a combination of the microsatellite markers D17Mit48, D17Mit57, D17Mit113, and D17Mit46 (Invitrogen) and SNP markers as shown on Figure 5C.

### Data analysis.

Data analysis was performed using the J/qtl software package (http://research.jax.org/faculty/churchill/software/Jqtl/index.html). The linkage between phenotyping and genotyping data was estimated by One QTL Genome Scan using the Imputation method and 10,000 permutations per scan. LOD scores above 3 were considered statistically significant (p < 0.05).

## Supporting Information

Table S1 SNP Markers Used in the Genome-Wide Mapping Study Marker ID are taken from [43]. Marker positions are according to NCBI Build 36.
(172 KB DOC)

Table S2 List of Primers Used in the Study Lowercase letters in the primer sequences indicate phosphorothioated bases.
(26 KB DOC)

Table S3 Markers and Positions for Mapping Interval 183.5–191.5 Mb and Detected Number of Crossovers Genomic positions in bp are according to NCBI Build 36.
(340 KB DOC)

## Abbreviations

Chr: Chromosome
CO: crossover
DSB: double-strand break
NCO: noncrossover
SNP: single nucleotide polymorphism
