# Supplementary material for: Trans-Regulation of Mouse Meiotic Recombination Hotspots by Rcr1
Source: PLoS Biol. 2009 Feb 17;7(2):e1000036. doi: 10.1371/journal.pbio.1000036 (PMC2642880; doi:10.1371/journal.pbio.1000036)
Supplement: Supplementary file 1 [file pbio.1000036.st001.doc]

**Supplementary Table 1. Markers used to genotype the mice in this study.**

| SNP ID/JAX Stock # | Chromosome | Position in bp (Build36) |
| --- | --- | --- |
| 01-009072542-M | 1 | 9013536 |
| 01-030151053-M | 1 | 29661843 |
| 01-043973192-M | 1 | 43394148 |
| 01-068512897-N | 1 | 67810119 |
| M22381_169_2 | 1 | 89035308 |
| 01-110152038-M | 1 | 111364114 |
| 01-127974550-M | 1 | 129016046 |
| 01-150091142-M | 1 | 150967689 |
| 01-168005000-P | 1 | 169814378 |
| 01-193173300-M | 1 | 193494553 |
| 02-004006715-M | 2 | 3954976 |
| 02-022708634-G | 2 | 22314077 |
| 02-041042651-M | 2 | 40416764 |
| 02-064172278-M | 2 | 63255540 |
| 02-083047417-M | 2 | 81944821 |
| 02-101920175-M | 2 | 100911329 |
| 02-119931810-M | 2 | 118676710 |
| 02-139603599-M | 2 | 138263384 |
| 02-159897340-M | 2 | 158705694 |
| 02-174167665-N | 2 | 172582591 |
| 02-178786326-G | 2 | 179503564 |
| 03-007561998-N | 3 | 7534689 |
| 03-026157475-M | 3 | 26523761 |
| 03-049202892-M | 3 | 49059524 |
| 03-069926915-G | 3 | 69155343 |
| 03-088155864-N | 3 | 87456454 |
| 03-106170268-G | 3 | 105971530 |
| 03-124203459-N | 3 | 123798104 |
| 03-138370314-M | 3 | 137805914 |
| 03-154247037-M | 3 | 153607529 |
| 04-003163167-M | 4 | 3649668 |
| M-01609_1 | 4 | 20161364 |
| 04-039199957-M | 4 | 39644013 |
| 04-057955407-M | 4 | 59130312 |
| 04-075869463-M | 4 | 78057952 |
| 04-097015585-M | 4 | 98721007 |
| 04-117238813-M | 4 | 118834992 |
| 04-137216461-M | 4 | 138853467 |
| 04-147973154-M | 4 | 151407011 |
| 05-008305854-N | 5 | 5587244 |
| 05-023890459-M | 5 | 24926478 |
| 05-044010971-M | 5 | 45342001 |
| 05-063053775-M | 5 | 64258964 |
| 05-081041323-M | 5 | 82582657 |
| 05-100871112-M | 5 | 103525303 |
| 05-118031699-M | 5 | 120242979 |
| 05-130950804-M | 5 | 132739200 |
| 05-150172420-M | 5 | 151185039 |
| 06-006933444-M | 6 | 7141467 |
| 06-024080365-M | 6 | 24082420 |
| 06-043835637-G | 6 | 43756420 |
| 06-064751205-M | 6 | 64136594 |
| 06-076285738-M | 6 | 75325059 |
| 06-098102692-N | 6 | 97249691 |
| 06-116730112-N | 6 | 115908655 |
| 06-136943848-M | 6 | 136551455 |
| 07-004201219-N | 7 | 4448294 |
| 07-014982749-M | 7 | 19100233 |
| 07-026273800-N | 7 | 35483177 |
| 07-043206744-N | 7 | 53453848 |
| 07-063201365-M | 7 | 73620881 |
| 07-082671949-N | 7 | 92808147 |
| 07-103184503-N | 7 | 113362440 |
| 07-120101158-G | 7 | 131053733 |
| 07-135024189-M | 7 | 144389200 |
| 08-003089774-M | 8 | 3189168 |
| 08-021183852-M | 8 | 23920179 |
| 08-041947937-M | 8 | 44278136 |
| 08-057901843-M | 8 | 61659280 |
| 08-074679809-N | 8 | 78023499 |
| 08-098079794-M | 8 | 101167992 |
| 08-115109448-M | 8 | 118025457 |
| 08-126038064-N | 8 | 128916688 |
| 09-003978896-N | 9 | 4060750 |
| 09-024686545-G | 9 | 24539827 |
| 09-040838924-M | 9 | 40389411 |
| 09-061963099-M | 9 | 61201832 |
| 09-082788952-M | 9 | 81656558 |
| 09-102158958-M | 9 | 101283122 |
| 09-122337931-M | 9 | 120768307 |
| 10-006360048-M | 10 | 3855880 |
| 10-023127952-M | 10 | 22876455 |
| 10-043132794-M | 10 | 42732201 |
| 10-060074134-G | 10 | 59831809 |
| 10-074905108-G | 10 | 74591762 |
| 10-093476458-N | 10 | 92695303 |
| 10-110502457-G | 10 | 110473608 |
| 10-128874353-N | 10 | 127612997 |
| 11-004367508-M | 11 | 4408733 |
| 11-023035271-N | 11 | 22905784 |
| 11-044333996-M | 11 | 43815998 |
| 11-063538723-N | 11 | 62913156 |
| 11-081316173-M | 11 | 80486866 |
| 11-100359477-N | 11 | 99396227 |
| 11-121181118-M | 11 | 120066584 |
| 12-003567042-M | 12 | 3701820 |
| 12-019163908-N | 12 | 25683951 |
| 12-037298090-M | 12 | 43814452 |
| 12-056129573-G | 12 | 62721558 |
| 12-074494300-M | 12 | 80727288 |
| 12-094386176-N | 12 | 100610611 |
| 12-112276179-M | 12 | 118496063 |
| 13-003966099-M | 13 | 4160601 |
| 13-023105764-M | 13 | 23703912 |
| 13-042824010-M | 13 | 43254273 |
| 13-061943220-M | 13 | 63128896 |
| 13-079289160-G | 13 | 83498304 |
| 13-098682853-M | 13 | 102393514 |
| 13-113057436-M | 13 | 116568685 |
| 14-004082624-M | 14 | 7586939 |
| 14-024063411-M | 14 | 27786220 |
| 14-037395207-M | 14 | 44497922 |
| 14-057298005-M | 14 | 65115960 |
| 14-072865727-G | 14 | 81045435 |
| 14-095233920-G | 14 | 103091632 |
| 14-109871050-M | 14 | 117514108 |
| 15-006448712-N | 15 | 6570318 |
| 15-020953071-M | 15 | 20827200 |
| 15-039906196-M | 15 | 39560443 |
| 15-063090495-M | 15 | 62470165 |
| 15-083914758-M | 15 | 82954168 |
| 15-103221933-M | 15 | 102073640 |
| 16-003310511-N | 16 | 3777477 |
| 16-022566879-G | 16 | 22720809 |
| 16-042056684-C | 16 | 42146192 |
| 16-064088849-C | 16 | 63734675 |
| 16-078751479-G | 16 | 78167427 |
| 16-093066825-C | 16 | 92251016 |
| 17-004147924-M | 17 | 5035660 |
| 17-025166339-M | 17 | 25710502 |
| 17-040218120-M | 17 | 40678690 |
| 17-061397942-G | 17 | 62659102 |
| 17-080241937-M | 17 | 80868471 |
| 17-092673068-N | 17 | 93503909 |
| 18-006845916-M | 18 | 6984866 |
| 18-027827579-M | 18 | 27823848 |
| 18-047938837-N | 18 | 47880237 |
| 18-065947946-M | 18 | 65735079 |
| 18-084751134-M | 18 | 84295967 |
| 19-007376322-N | 19 | 3455121 |
| 19-018239318-M | 19 | 18661904 |
| 19-039967462-M | 19 | 40316900 |
| 19-060030696-N | 19 | 60023949 |
| X-016114535-G | X | 17469781 |
| X-029230990-G | X | 39226538 |
| X-041569348-G | X | 53881296 |
| X-060478145-M | X | 73717295 |
| X-082721065-G | X | 94126733 |
| X-095788781-G | X | 106808631 |
| X-111633440-M | X | 127594040 |
| X-131785813-G | X | 147238083 |
| X-143466659-M | X | 161659424 |
