# Supplementary material for: Trans-Regulation of Mouse Meiotic Recombination Hotspots by Rcr1
Source: PLoS Biol. 2009 Feb 17;7(2):e1000036. doi: 10.1371/journal.pbio.1000036 (PMC2642880; doi:10.1371/journal.pbio.1000036)
Supplement: Supplementary file 2 [file pbio.1000036.st002.doc]

| Primer | Sequence |
| --- | --- |
| 184253-F1B  184253-R1C  184253-F2B  184253-R2C  187726-F1B  187726-R1C  187726-F2B  187726-R2C  189000F1B  189000R1C  189000F2B  189000R2C | 5’- CCGGTTTCAGTTTCCAGagc-3’  5’-GTCGGCTGGGAAGAaga-3’  5’-CTGCCCAGGCTATCTAGGTacg-3’  5’-GTTTTGTTTTTCCCTTTATACATCTCCTacc-3’  5’- CCTCACAAATGCACCCcat-3’  5’- TCCAAAACAGGAAGAGACTTACAAgat-3’  5’- AGAGTAAATGTGTTCCTGTGTTATgtg-3’  5’- CCATCATGTATAGAGAATACATGCTAATagg-3’  5’-GGGAAAGATAAACATAAATGACAGTAAAGGttc-3’  5’- GAAACACAATGGGAGCAccg-3’  5’- CACCAAGTTGGCTCcga-3’  5’- TTCACACACTCCTGAGCAAcaa-3’ |

**Supplementary Table 2. List of primers used in the study.** The last three nucleotides in the primers sequence are with small letters representing PTO-modification.
