# Supplementary material for: Trans-Regulation of Mouse Meiotic Recombination Hotspots by Rcr1
Source: PLoS Biol. 2009 Feb 17;7(2):e1000036. doi: 10.1371/journal.pbio.1000036 (PMC2642880; doi:10.1371/journal.pbio.1000036)
Supplement: Supplementary file 3 [file pbio.1000036.st003.doc]

**Supplementary Table 3. Markers and positions for mapping interval 183.5-191.5 Mb and detected number of crossovers.**

|  |  | B6xB6.CAST-1T | |
| --- | --- | --- | --- |
| Marker ID | Marker Position (bp) | Female COs | Male COs |
| NES10634618 | 183469141 | 1 | 0 |
| NES12844841 | 183837556 | 0 | 0 |
| 01-182362000-P | 184115513 | 0 | 0 |
| D1P182389000sslp | 184142098 | 0 | 0 |
| D1P182444000sslp | 184197098 | 0 | 0 |
| NES15853911 | 184199041 | 0 | 0 |
| 01-182510000-P | 184261988 | 0 | 0 |
| 01-182577000-P | 184328327 | 4 | 2 |
| NES15859292 | 184408640 | 0 | 0 |
| 01-182666000-P | 184416062 | 0 | 0 |
| 01-182835000-P | 184586932 | 0 | 0 |
| 01-183035000-P | 184773266 | 0 | 0 |
| NES15858130 | 184807218 | 6 | 7 |
| NES15858012 | 185219234 | 7 | 7 |
| 01-183336000-P | 185404275 | 0 | 0 |
| 01-183475000-P | 185543298 | 0 | 0 |
| NES15852051 | 185566246 | 0 | 0 |
| NES15851399 | 185589068 | 0 | 0 |
| NES15850498 | 185629664 | 0 | 0 |
| NES15850036 | 185650733 | 1 | 0 |
| NES15855481 | 185679806 | 1 | 0 |
| NES15854695 | 185725738 | 0 | 0 |
| NES15853778 | 185776256 | 0 | 0 |
| NES14450962 | 185869522 | 0 | 0 |
| NES14450038 | 185900602 | 3 | 4 |
| NES14449712 | 186061449 | 0 | 1 |
| 01-183996000-P | 186071894 | 0 | 0 |
| 01-184008000-P | 186083609 | 0 | 0 |
| 01-184228000-P | 186293245 | 0 | 0 |
| NES15850925 | 186303690 | 0 | 0 |
| 01-184251000-P | 186315877 | 0 | 0 |
| 01-184254000-P | 186318716 | 0 | 0 |
| 01-184263000-P | 186327474 | 0 | 0 |
| NES15858934 | 186366646 | 0 | 0 |
| NES12819988 | 186375404 | 0 | 0 |
| 01-184327000-P | 186391407 | 0 | 0 |
| 01-184336000-P | 186400165 | 0 | 0 |
| NES12811872 | 186436634 | 0 | 0 |
| NES15858629 | 186458296 | 0 | 0 |
| NES12819988 | 186519007 | 1 | 0 |
| 01-184462000-P | 186526801 | 0 | 0 |
| NES12811872 | 186649934 | 0 | 1 |
| 01-184709000-P | 186772844 | 1 | 0 |
| 01-186311955-M | 186902281 | 0 | 1 |
| NES10638946 | 187147794 | 4 | 0 |
| 01-185962173-P | 187358738 | 0 | 0 |
| 01-185337000-P | 187399917 | 0 | 0 |
| NES10658870 | 187445960 | 0 | 0 |
| 01-185423000-P | 187485664 | 0 | 4 |
| NES10655318 | 187507064 | 0 | 0 |
| 01-185477000-P | 187539882 | 0 | 0 |
| NES10651090 | 187587911 | 3 | 0 |
| 01-185683000-P | 187745315 | 0 | 0 |
| 01-185732000-P | 187794310 | 0 | 0 |
| NES10650466 | 187827793 | 2 | 0 |
| NES10650390 | 187830619 | 0 | 0 |
| NES10650115 | 187833914 | 0 | 0 |
| NES10647579 | 187877470 | 0 | 0 |
| 01-185849000-P | 187911310 | 0 | 0 |
| NES10645110 | 187931829 | 0 | 0 |
| NES10644536 | 187952025 | 0 | 0 |
| 01-185982000-P | 188045382 | 0 | 0 |
| 01-186329000-P | 188386468 | 2 | 0 |
| 01-187966083-M | 188534548 | 0 | 0 |
| 01-186365000-P | 188558147 | 0 | 0 |
| 01-186372000-P | 188565147 | 0 | 0 |
| 01-186383600-P | 188576747 | 0 | 0 |
| 01-186387200 | 188580376 | 0 | 0 |
| 01-186389000-P | 188582176 | 1 | 2 |
| NES12887626 | 188654120 | 1 | 3 |
| NES12911581 | 188732698 | 1 | 0 |
| 01-187400874-P | 188783959 | 0 | 0 |
| NES10635264 | 189005806 | 1 | 0 |
| 01-187700736-P | 189085232 | 0 | 1 |
| NES10630644 | 189108459 | 0 | 0 |
| NES10660147 | 189131648 | 2 | 2 |
| NES10659009 | 189155633 | 0 | 0 |
| NES10657958 | 189179092 | 0 | 0 |
| NES10656804 | 189202526 | 0 | 1 |
| 01-187180000-P | 189234945 | 0 | 0 |
| NES10654608 | 189249110 | 0 | 3 |
| NES10653422 | 189271012 | 0 | 0 |
| NES10652097 | 189294901 | 0 | 1 |
| NES10651493 | 189304399 | 0 | 0 |
| NES10650600 | 189326310 | 0 | 0 |
| NES10649715 | 189339853 | 1 | 1 |
| 01-188000435-P | 189382638 | 3 | 0 |
| NES10646969 | 189400769 | 0 | 0 |
| 01-187450000-P | 189503203 | 0 | 0 |
| 01-187521000-P | 189574849 | 0 | 0 |
| NES15860686 | 189583988 | 5 | 2 |
| NES15860668 | 189584841 | 0 | 0 |
| 01-187393000-P | 189586000 | 0 | 0 |
| 01-187547000-P | 189599381 | 0 | 0 |
| 01-187547318-P | 189599699 | 0 | 0 |
| NES15860024 | 189620118 | 0 | 0 |
| 01-187622000-P | 189673847 | 2 | 2 |
| 01-187676000-P | 189728900 | 1 | 0 |
| 01-187721000-P | 189773317 | 0 | 0 |
| NES14455879 | 189778759 | 0 | 0 |
| 01-187729000-P | 189782023 | 0 | 0 |
| 01-187732000-P | 189785048 | 3 | 4 |
| 01-187735000-P | 189787471 | 0 | 0 |
| NES14454790 | 189830881 | 2 | 0 |
| 01-188500788-P | 189881909 | 3 | 5 |
| 01-187950000-P | 190001088 | 5 | 9 |
| NES15859505 | 190055699 | 0 | 0 |
| NES15859137 | 190078356 | 0 | 0 |
| NES15858760 | 190103487 | 0 | 0 |
| 01-188750734-P | 190128804 | 1 | 0 |
| NES15857199 | 190179787 | 0 | 0 |
| NES15856731 | 190204311 | 0 | 1 |
| NES15855555 | 190270379 | 0 | 0 |
| NES15852908 | 190377290 | 0 | 1 |
| NES14453751 | 190430334 | 0 | 0 |
| 01-190055476-M | 190601677 | 0 | 0 |
| NES14450252 | 190620272 | 0 | 0 |
| NES14449905 | 190651195 | 0 | 1 |
| NES14449741 | 190670271 | 0 | 0 |
| NES14449761 | 190672710 | 0 | 0 |
| NES14448747 | 190729398 | 0 | 0 |
| NES14449035 | 190732070 | 0 | 0 |
| NES14448656 | 190734993 | 0 | 0 |
| NES10657138 | 190737384 | 0 | 0 |
| NES10656867 | 190740181 | 0 | 7 |
| NES10656906 | 190742791 | 0 | 2 |
| NES10655769 | 190787455 | 0 | 0 |
| NES10654243 | 190817421 | 1 | 0 |
| NES10652675 | 190844141 | 0 | 0 |
| NES10651934 | 190858211 | 0 | 0 |
| NES10650887 | 190878444 | 2 | 2 |
| NES10648320 | 190922493 | 0 | 1 |
| NES10647330 | 190942727 | 0 | 0 |
| NES10645775 | 190971711 | 0 | 0 |
| NES10645310 | 190983991 | 0 | 0 |
| NES10643254 | 191027377 | 0 | 0 |
| NES10643133 | 191030400 | 0 | 0 |
| 01-189203000-P | 191048377 | 0 | 0 |
| NES10641259 | 191074466 | 0 | 0 |
| NES10639424 | 191123969 | 0 | 0 |
| NES10638662 | 191142448 | 0 | 0 |
| NES10637350 | 191191973 | 0 | 3 |
| 01-189394000-P | 191231287 | 2 | 1 |
| NES10641321 | 191381096 | 0 | 0 |
| NES10640089 | 191407866 | 0 | 0 |
| NES10638252 | 191440196 | 0 | 1 |
| NES10637972 | 191444998 | 10 | 6 |
| NES10637793 | 191448010 | 0 | 0 |
| NES10637315 | 191456399 | 0 | 0 |
| NES10635761 | 191486051 | 0 | 0 |
| NES10635384 | 191493757 | 0 | 1 |
| NES10633126 | 191541510 | 0 | 0 |
|  |  |  |  |
|  |  |  |  |
|  |  |  |  |
|  | B6xCAST |  |  |
| Marker ID | Marker Position (bp) | Female COs | Male COs |
| NES10634618 | 183469141 | 0 | 1 |
| NES12844841 | 183837556 | 4 | 5 |
| 01-182362000-P | 184115513 | 1 | 1 |
| D1P182389000sslp | 184142098 | 0 | 0 |
| D1P182444000sslp | 184197098 | 25 | 23 |
| NES15853911 | 184199041 | 0 | 0 |
| 01-182510000-P | 184261988 | 0 | 0 |
| 01-182577000-P | 184328327 | 0 | 0 |
| NES15859292 | 184408640 | 0 | 0 |
| 01-182666000-P | 184416062 | 0 | 0 |
| 01-182835000-P | 184586932 | 1 | 2 |
| 01-183035000-P | 184773266 | 0 | 0 |
| NES15858130 | 184807218 | 0 | 0 |
| NES15858012 | 185219234 | 21 | 18 |
| 01-183336000-P | 185404275 | 0 | 0 |
| 01-183475000-P | 185543298 | 0 | 0 |
| NES15852051 | 185566246 | 13 | 23 |
| NES15851399 | 185589068 | 0 | 0 |
| NES15850498 | 185629664 | 0 | 0 |
| NES15850036 | 185650733 | 1 | 0 |
| NES15855481 | 185679806 | 8 | 4 |
| NES15854695 | 185725738 | 0 | 2 |
| NES15853778 | 185776256 | 0 | 4 |
| NES14450962 | 185869522 | 2 | 0 |
| NES14450038 | 185900602 | 2 | 3 |
| NES14449712 | 186061449 | 5 | 0 |
| 01-183996000-P | 186071894 | 0 | 0 |
| 01-184008000-P | 186083609 | 0 | 0 |
| 01-184228000-P | 186293245 | 0 | 0 |
| NES15850925 | 186303690 | 0 | 0 |
| 01-184251000-P | 186315877 | 17 | 91 |
| 01-184254000-P | 186318716 | 0 | 0 |
| 01-184263000-P | 186327474 | 0 | 0 |
| NES15858934 | 186366646 | 0 | 0 |
| NES12819988 | 186375404 | 0 | 0 |
| 01-184327000-P | 186391407 | 0 | 0 |
| 01-184336000-P | 186400165 | 1 | 13 |
| NES12811872 | 186436634 | 0 | 0 |
| NES12819988 | 186519007 | 0 | 0 |
| 01-184462000-P | 186526801 | 0 | 0 |
| NES12811872 | 186649934 | 1 | 1 |
| 01-184709000-P | 186772844 | 5 | 2 |
| 01-186311955-M | 186902281 | 0 | 0 |
| NES10638946 | 187147794 | 3 | 2 |
| 01-185962173-P | 187358738 | 0 | 0 |
| 01-185337000-P | 187399917 | 0 | 5 |
| NES10658870 | 187445960 | 0 | 5 |
| 01-185423000-P | 187485664 | 0 | 10 |
| NES10655318 | 187507064 | 0 | 1 |
| 01-185477000-P | 187539882 | 0 | 0 |
| NES10651090 | 187587911 | 0 | 0 |
| 01-185683000-P | 187745315 | 0 | 1 |
| 01-185732000-P | 187794310 | 0 | 0 |
| NES10650466 | 187827793 | 26 | 25 |
| NES10650390 | 187830619 | 0 | 1 |
| NES10650115 | 187833914 | 0 | 0 |
| NES10647579 | 187877470 | 0 | 1 |
| 01-185849000-P | 187911310 | 6 | 1 |
| NES10645110 | 187931829 | 2 | 0 |
| NES10644536 | 187952025 | 4 | 0 |
| 01-185982000-P | 188045382 | 0 | 3 |
| 01-186329000-P | 188386468 | 1 | 0 |
| 01-187966083-M | 188534548 | 1 | 0 |
| 01-186365000-P | 188558147 | 0 | 0 |
| 01-186372000-P | 188565147 | 2 | 0 |
| 01-186383600-P | 188576747 | 0 | 0 |
| 01-186387200 | 188580376 | 2 | 18 |
| 01-186389000-P | 188582176 | 0 | 0 |
| NES12887626 | 188654120 | 3 | 1 |
| NES12911581 | 188732698 | 0 | 0 |
| 01-187400874-P | 188783959 | 1 | 0 |
| NES10635264 | 189005806 | 0 | 0 |
| 01-187700736-P | 189085232 | 0 | 0 |
| NES10656804 | 189202526 | 0 | 0 |
| 01-187180000-P | 189234945 | 0 | 0 |
| 01-188000435-P | 189382638 | 1 | 0 |
| NES10646969 | 189400769 | 0 | 0 |
| 01-187450000-P | 189503203 | 0 | 0 |
| 01-187521000-P | 189574849 | 0 | 0 |
| NES15860686 | 189583988 | 10 | 21 |
| NES15860668 | 189584841 | 0 | 0 |
| 01-187393000-P | 189586000 | 1 | 0 |
| 01-187547000-P | 189599381 | 0 | 0 |
| 01-187547318-P | 189599699 | 0 | 0 |
| NES15860024 | 189620118 | 0 | 0 |
| 01-187622000-P | 189673847 | 0 | 0 |
| 01-187676000-P | 189728900 | 10 | 15 |
| 01-187721000-P | 189773317 | 0 | 1 |
| NES14455879 | 189778759 | 19 | 41 |
| 01-187729000-P | 189782023 | 0 | 0 |
| 01-187732000-P | 189785048 | 14 | 52 |
| 01-187735000-P | 189787471 | 5 | 11 |
| NES14454790 | 189830881 | 0 | 0 |
| 01-188500788-P | 189881909 | 3 | 6 |
| 01-187950000-P | 190001088 | 1 | 1 |
| 01-188750734-P | 190128804 | 2 | 0 |
| NES15857199 | 190179787 | 13 | 1 |
| NES15856731 | 190204311 | 0 | 0 |
| NES15855555 | 190270379 | 0 | 0 |
| NES15852908 | 190377290 | 0 | 0 |
| NES14453751 | 190430334 | 0 | 0 |
| 01-190055476-M | 190601677 | 7 | 8 |
| NES14450252 | 190620272 | 1 | 0 |
| NES14449905 | 190651195 | 0 | 0 |
| NES14449741 | 190670271 | 0 | 0 |
| NES14449761 | 190672710 | 0 | 0 |
| NES14448747 | 190729398 | 0 | 0 |
| NES14449035 | 190797982 | 0 | 0 |
| NES10652675 | 190844141 | 1 | 1 |
| NES10651934 | 190858211 | 0 | 0 |
| NES10650887 | 190878444 | 0 | 3 |
| NES10648320 | 190922493 | 0 | 2 |
| NES10647330 | 190942727 | 0 | 1 |
| NES10645775 | 190971711 | 0 | 0 |
| NES10645310 | 190983991 | 0 | 0 |
| NES10643254 | 191027377 | 11 | 22 |
| NES10643133 | 191030400 | 0 | 0 |
| 01-189203000-P | 191048377 | 0 | 8 |
| NES10641259 | 191074466 | 0 | 0 |
| NES10639424 | 191123969 | 0 | 0 |
| NES10638662 | 191142448 | 0 | 0 |
| NES10637350 | 191191973 | 2 | 3 |
| 01-189394000-P | 191231287 | 3 | 7 |
| NES10641321 | 191381096 | 2 | 0 |
| NES10633126 | 191541510 | 5 | 3 |
